# Filling knowledge gaps related to AmpC-dependent β-lactam resistance in *Enterobacter cloacae*

**DOI:** 10.1038/s41598-023-50685-1

**Published:** 2024-01-02

**Authors:** Isabel M. Barceló, María Escobar-Salom, Elena Jordana-Lluch, Gabriel Torrens, Antonio Oliver, Carlos Juan

**Affiliations:** 1 Health Research Institute of the Balearic Islands (IdISBa), 07010 Palma, Spain; 2 Microbiology Department, University Hospital Son Espases (HUSE), 07010 Palma, Spain; 3 Centro de Investigación Biomédica en Red, Área Enfermedades Infecciosas (CIBERINFEC), Instituto de Salud Carlos III, 28029 Madrid, Spain; 4 Department of Molecular Biology and Laboratory for Molecular Infection Medicine Sweden (MIMS), Umeå Centre for Microbial Research (UCMR), Umeå University, 901 87 Umeå, Sweden

**Keywords:** Microbiology, Medical research

## Abstract

*Enterobacter cloacae* starred different pioneer studies that enabled the development of a widely accepted model for the peptidoglycan metabolism-linked regulation of intrinsic class C cephalosporinases, highly conserved in different Gram-negatives. However, some mechanistic and fitness/virulence-related aspects of *E. cloacae* choromosomal AmpC-dependent resistance are not completely understood. The present study including knockout mutants, β-lactamase cloning, gene expression analysis, characterization of resistance phenotypes, and the *Galleria mellonella* infection model fills these gaps demonstrating that: (i) AmpC enzyme does not show any collateral activity impacting fitness/virulence; (ii) AmpC hyperproduction mediated by *ampD* inactivation does not entail any biological cost; (iii) alteration of peptidoglycan recycling alone or combined with AmpC hyperproduction causes no attenuation of *E. cloacae* virulence in contrast to other species; (iv) derepression of *E. cloacae* AmpC does not follow a stepwise dynamics linked to the sequential inactivation of AmpD amidase homologues as happens in *Pseudomonas aeruginosa*; (v) the enigmatic additional putative AmpC-type β-lactamase generally present in *E. cloacae* does not contribute to the classical cephalosporinase hyperproduction-based resistance, having a negligible impact on phenotypes even when hyperproduced from multicopy vector. This study reveals interesting particularities in the chromosomal AmpC-related behavior of *E. cloacae* that complete the knowledge on this top resistance mechanism.

## Introduction

The growing levels of antimicrobial resistance in bacterial opportunistic pathogens pose one of the greatest threats for public health in the twenty-first century^1^. This phenomenon is especially relevant in species that stand out because of their successful nosocomial dissemination, virulence, and derived high morbidity-mortality rates, mostly gathered within the ESKAPE group^2^. Among its Gram-negative members, *Pseudomonas aeruginosa* and *Enterobacter cloacae* predominantly base β-lactam resistance on their intrinsic chromosomal class C inducible cephalosporinase (AmpC), a fearsome weapon that has already displayed the mutation-driven capacity to counteract the most recent β-lactam-β-lactamase inhibitor combinations^3^. Therefore, AmpC β-lactamases pose one of the top resistance mechanisms that must be considered to develop classic, anti-virulence, and/or resistance-breaking strategies^4,5^.

*Pseudomonas aeruginosa* is probably the species in which chromosomal AmpC β-lactamase has been most thoroughly characterized from all the perspectives^6–16^. The regulatory mechanisms of *P. aeruginosa* AmpC are thought to be highly equivalent to those of certain *Enterobacteriaceae* species, namely *E. cloacae* and *Citrobacter freundii*, used in the pioneer studies characterizing the peptidoglycan metabolism-linked regulation of intrinsic class C cephalosporinases, proved to be under the control of LysR-type transcriptional regulators (AmpR)^17–22^. Briefly, depending on the fragments of soluble peptidoglycan (muropeptides) released, internalized into cytosol for recycling, and finally bound to AmpR, this acquires different conformations that either promote the transcription of *ampC* (e.g., during an inducer β-lactam challenge) or keep it repressed at basal levels (when the regulator binds to certain newly synthesized peptidoglycan precursors). AmpR performs this role by interacting with the intergenic region present between its own encoding gene and *ampC*, which are divergently transcribed^11,23^. AmpR causes bending/relaxation of this intergenic DNA, conditioning interactions with RNA polymerases, which accounts for the repressed/promoted transcription of the β-lactamase^11,23^. This mechanism has been interpreted as a sentinel strategy to sense increased peptidoglycan damage and respond with a transiently boosted production of the β-lactamase to neutralize β-lactam aggression^11,22^. The abovementioned pioneer studies in *Enterobacteriaceae* revealed some mutational pathways leading to a constitutive AmpC hyperproduction and increased resistance, thus not only affecting hydrolysable inducer β-lactams^21,22^. These *classical* mutations were those disabling the function of the indirect AmpC repressor AmpD amidase, or specific amino acid changes providing a constitutive activator conformation of AmpR itself^17–22,24–28^.

Although the AmpR-AmpC systems of *Enterobacteriaceae* and *P. aeruginosa* are considered highly homologous^7,11^, some differences and knowledge gaps still exist regarding the former. For instance, in *P. aeruginosa* some additional targets leading to AmpC hyper-production through inactivating mutations in genes such as *mpl* have been described^29,30^, but their potential impact on *Enterobacteriaceae* has been barely approached. Moreover, the mutational inactivation of *dacB* (encoding the Penicillin Binding Protein 4, PBP4) is probably the most common cause of AmpC hyperproduction in *P. aeruginosa*^10^, whereas in *Enterobacteriaceae* its impact seems more limited and variable depending on the species^31,32^. Meanwhile, in *P. aeruginosa* an interesting stepwise model to obtain increasing levels of AmpC production and resistance parallel to the sequential inactivation of the amidase homologues AmpD, AmpDh2, and AmpDh3 was described^9^. However, the potential existence of a similar model in AmpC-harboring *Enterobacteriaceae* (which would have only two steps since they harbor a unique AmpD additional homologue), has not been investigated. In fact, different AmpC hyperproduction phenotypes have been described in *Enterobacteriaceae* linked to different mutations in *ampD,* but an unequivocal cause-effect relatedness has not been established. In other words, in some cases the *ampD* mutation caused a total derepression of AmpC, whereas in others, it caused a partially de-repressed phenotype (further inducible)^21,33–37^. Therefore, the never explored potential influence of the additional AmpD homologue cannot be ruled out. Besides, although a putative additional class C β-lactamase gene has been almost uniformly found in the genomes of *E. cloacae* and which seemingly does not take part in the basal resistance phenotype^31^, its potential contribution to increase the resistance output in a context of mutation-driven classical AmpC hyperproduction has not been investigated. Moreover, the potential impact of hyperproducing the mentioned putative β-lactamase per se on the phenotype has not been approached as yet.

Finally, in contrast with other species^12,14,16^*,* the interplay between chromosomal AmpC-dependent resistance, its peptidoglycan metabolism-related regulation, and the potentially associated fitness/virulence costs is an almost unexplored field in *Enterobacteriaceae*. Only some specific amino acid variants in AmpR have been characterized in this regard, suggesting insignificant associated costs^38^. Conversely, it is still unknown whether AmpC β-lactamase hyper-production per se, and/or achieved through typical mutational pathways could dampen virulence, and/or whether the alteration of peptidoglycan recycling itself could enhance this outcome, as happens in *P. aeruginosa*^14,16,39^.

Attempting to fill these AmpC regulatory/virulence-related knowledge gaps in *E. cloacae* was our general objective. Our results help to understand the particular nature of this mechanism in this species and will hopefully be useful for the development of strategies intended to disable it.

## Results and discussion

### Basic regulatory features and connections with peptidoglycan recycling and virulence of *E. cloacae* AmpC

**Figure 1:**
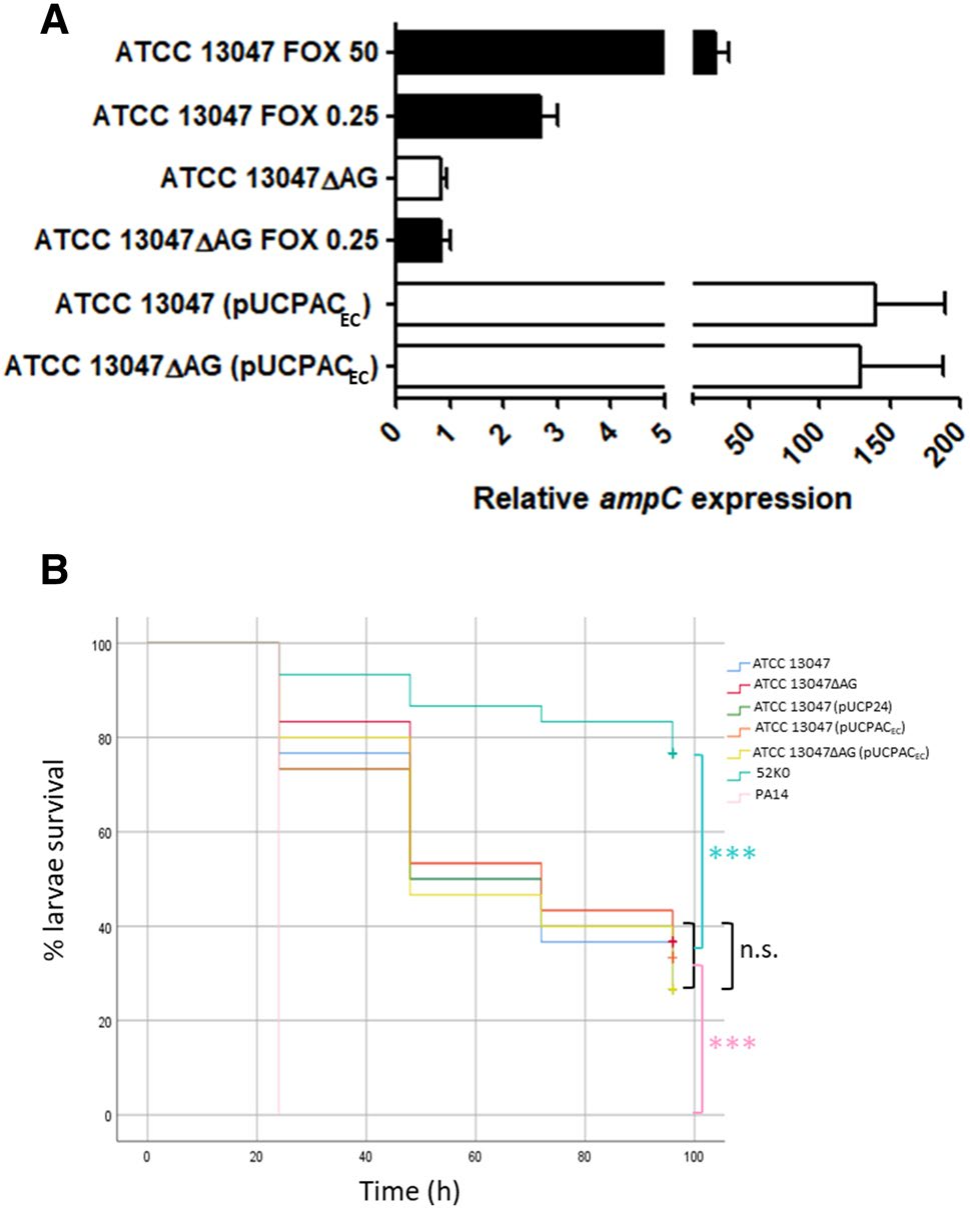
(**A**) Relative quantification of the *ampC* (ECL_00553) mRNA in the indicated strains, considering the wildtype ATCC 13047 strain basal level as 1 and using the *rpoB* gene for normalization. Horizontal columns represent the mean values from experimental replicates, whereas the error bars correspond to the standard deviations (SD) (linear scale). White columns correspond to mRNA extracted in basal situation, whereas black ones correspond to induction conditions with cefoxitin (FOX) at the indicated concentrations (mg/l). (**B**) Virulence behavior of the indicated strains in the *G. mellonella* infection model. The proportion of surviving larvae after infection with 5E^6^ CFU/worm at the controlled time points (24, 48, 72, 96 h) is shown. Statistical analysis (Kaplan–Meier curves and log-rank test) was performed for pairwise comparisons between all strains. The two controls used were *P. aeruginosa* PA14 (high virulence) and *K. pneumoniae* 52K0 (low virulence). ***p value < 0.001; *p value < 0.05; *n.s.* not significant, i.e. p value > 0.05.

As previously demonstrated^17,31^, inactivation of *E. cloacae* AmpG permease abolished the AmpC inducibility and dramatically impaired the level of resistance to hydrolysable inducer β-lactams such as cefoxitin (Fig. 1A, Table 1). Whereas cefoxitin at 50 and 0.25 mg/l caused an increase in the *ampC* mRNA of ca. 25 and threefold respectively in the wildtype strain compared to basal situation, the levels of *ampC* expression in ATCC 13047ΔAG and the same mutant induced with cefoxitin 0.25 mg/l (1/4–1/8 of MIC, concentrations previously shown to be effective for induction^40,41^) were very similar to those of ATCC 13047 in non-inducing conditions (Fig. 1A). These results are explained by the role that AmpG permease plays for the cytosolic internalization of soluble muropeptides enabling peptidoglycan recycling on one hand, and AmpR activator conformation promoting *ampC* hyperexpression on the other^12,22^.

**Table 1:** Minimum inhibitory concentrations (MICs) of selected β-lactam drugs against the specified *E. cloacae/E. coli* strains.

Strain	MIC (mg/l)									
	AMP	AMX/CLA	FOX	CTX	CAZ	FEP	PIP/TAZ	ATM	TOL/TAZ	CAZ/AVI
*E. cloacae* ATCC 13047	> 256	> 256	> 256	0.38	0.38	0.032	1.5	0.5	0.38	0.19
*E. cloacae* ATCC 13047ΔAG	2	3	1.5	0.032	0.094	< 0.016	0.125	0.047	0.19	0.064
*E. cloacae* ATCC 13047 (pUCPAC_EC_)	> 256	> 256	> 256	8	8	0.064	8	4	1.5	0.25
*E. cloacae* ATCC 13047ΔAG (pUCPAC_EC_)	> 256	> 256	> 256	4	3	0.047	4	3	0.5	0.094
*E. cloacae* ATCC 13047 (pUCP24)	> 256	> 256	> 256	0.38	0.38	0.032	1.5	0.38	0.38	0.19
*E. cloacae* ATCC 13047ΔAG (pUCP24)	1.5	3	1.5	0.032	0.064	< 0.016	0.125	0.047	0.19	0.064
*E. coli* XL1 Blue	8	3	16	0.064	0.125	0.064	1	0.125	0.25	0.125
*E. coli* XL1 Blue pUCPECL_03254	8	3	16	0.125	0.19	0.094	1	0.125	0.25	0.19
*E. cloacae* ATCC 13047	> 256	> 256	> 256	0.38	0.5	0.032	1.5	0.5	0.38	0.19
*E. cloacae* ATCC 13047 pUCPECL_03254	> 256	> 256	> 256	0.5	0.75	0.032	1.5	0.5	0.5	0.25

*AMP* ampicillin, *AMX/CLA* amoxicillin/clavulanate, *FOX* cefoxitin, *CTX* cefotaxime, *CAZ* ceftazidime, *FEP* cefepime, *PIP/TAZ* piperacillin-tazobactam, *ATM* aztreonam, *TOL/TAZ* ceftolozane-tazobactam, *CAZ/AVI* ceftazidime-avibactam.

The balance between resistance and virulence is a topic increasingly approached as a potential source of targets useful for anti-virulence and/or resistance-breaking strategies^42–46^. In this context, it has been shown that the production of certain β-lactamases (e.g. some OXA-2-derived variants) per se entails dramatic virulence attenuations in *P. aeruginosa*, presumably through residual activities of the produced enzyme on peptidoglycan biology^16,23,39,47–49^. Similar data had been published before for other Gram-negative species and intrinsic/acquired β-lactamases^14,50–54^. Thus, in this study, we wanted to decipher whether the hyperproduction of AmpC per se, regardless of its underlying mechanisms, could impair *E. cloacae* pathogenic power in a homologous way, likely through enzymatic collateral impacts on peptidoglycan biology. To avoid parallel effects linked to mutation-driven mechanisms, we obtained AmpC hyperproduction through the multicopy plasmid pUCPAC_EC_ (*ampC* mRNA relative amount ca. 140-fold compared to wildtype basal, Fig. 1A), whose expression had no negative effects on either ATCC 13047 or ATCC 13047ΔAG capacities for *Galleria mellonella* killing (log rank test *p* values > 0.05, Fig. 1B). Consequently, neither AmpC hypeproduction itself [ATCC 13047 (pUCPAC_EC_)], nor peptidoglycan recycling impairment through *ampG* inactivation (ATCC 13047ΔAG), nor the combination of both facts had any effects on *E. cloacae* virulence (Fig. 1B). These results are in accordance with previous data showing that the cloned AmpC of *E. cloacae*, when expressed in *E. coli* and *P. aeruginosa,* did not alter their motility or biofilm formation capacity in contrast with other class A/D enzymes^51^. Conversely, our results would not be in line with some recent data in *P. aeruginosa* showing that the alteration of peptidoglycan recycling itself caused a significant virulence attenuation in *G. mellonella* and murine models^15,16,39^. In fact, also in *P. aeruginosa*, the combination of peptidoglycan recycling blockade with the production of specific β-lactamases (including intrinsic AmpC, but also other acquired ones) significantly accentuated the biological cost associated with each feature separately, representing a clear difference from the present results regarding the intrinsic cephalosporinase of *E. cloacae*^16,39^. This supports the previous idea of significant peptidoglycan metabolism particularities depending on the species, which could therefore entail different impacts for virulence associated to recycling-altered scenarios^55^. Hence, our results suggest that *E. cloacae* displays little susceptibility to peptidoglycan metabolism alterations as a cause of virulence attenuation, which would disable the blockade of cell wall recycling as a valid anti-virulence strategy against *E. cloacae*, in contrast to *P. aeruginosa*^15^. This feature would be common to another *Enterobacteriaceae* species, *Salmonella enterica*, in which AmpG disruption was proved to have no negative impact for fitness/virulence either^56^. Conversely, as demonstrated by the lack of induction in the AmpG-defective strain (Table 1, Fig. 1A), blockade of peptidoglycan recycling continues to be a valid idea to disable the AmpC-dependent resistance in *E. cloacae*^42^.

In conclusion it can be deduced that, although β-lactamases obviously share the capacity for β-lactam ring hydrolysis, their potential effects on the polymeric and/or soluble peptidoglycan may not be uniform^11,16,23,39,45^. This could explain why β-lactamase production displays a wide range of different impacts on virulence depending on the enzyme. These effects would also depend on the species and on the respective peptidoglycan metabolism particularities^11,16,23,39,45^. Therefore, in order to find therapeutic targets in the virulence-resistance interplay and in the specific field of β-lactamases, all these variables should be considered.

### Mutation-driven AmpC hyperproduction in *E. cloacae*: resistance and virulence implications of the archetypical mechanism

Through seeding different dilutions of *E. cloacae* ATCC 13047 overnight liquid cultures on cefotaxime-supplemented LB plates, plenty of spontaneous resistant colonies were obtained with an estimated frequency between 5E^−5^ and 5E^−6^, a range in accordance with previous results^25,57,58^. The expected resistance mechanism was intrinsic AmpC hyperproduction, since cefotaxime has been widely used to select this type of mutants^58^. We randomly picked five resistant colonies, and in line with previous evidence^31,32^, the basis for their phenotype was the inactivation of AmpD amidase through different mutations (Table 2), as determined by sequencing. In this regard, although some specific amino acid changes providing a constitutive activator conformation of AmpR have also been described as a cause of AmpC hyperproduction^21,24–26^, this mechanism appears less frequently than *ampD* inactivation because of probabilistic reasons: specific amino acid changes in few AmpR positions vs any mechanism of inactivation, such as frameshift mutations, stop codons, or specific amino acid changes in AmpD^21,24,59^. In accordance with our selected mutants, *ampD* inactivation has been considered by pioneer and more recent studies with wide collections of clinical isolates as the archetypical cause of AmpC hyperproduction in *E. cloacae* and closely related species^21,25,27,28,31,32^.

**Table 2:** MICs of different β-lactams against the indicated *E. cloacae* wildtype strain, spontaneous AmpD-defective mutants and ECL_02804 KO mutants constructed in the laboratory.

Strain	MICs (mg/l)							*ampD* mutation
	CTX	CAZ	FEP	PIP/TAZ	ATM	TOL/TAZ	CAZ/AVI	
ATCC 13047	0.38	0.38	0.032	1.5	0.5	0.38	0.19	NA
ATCC 13047ΔAD	32	48	0.5	48	12	6	0.25	Deletion of nt 432
ATCC 13047ΔAD-b	32	64	0.5	48	12	6	0.25	Deletion of 4 nts (183–186)
ATCC 13047ΔAD-c	32	48	0.5	48	12	6	0.25	Deletion of nt 279
ATCC 13047ΔAD-d	32	48	0.5	96	12	4	0.25	Deletion of 2 nts (276–277)
ATCC 13047ΔAD-e	48	48	0.5	48	24	4	0.25	C → T change (nt 385): stop codon
ATCC 13047 Δ02804	0.38	0.38	0.032	1	0.25	0.5	0.19	NA
ATCC 13047ΔAD Δ02804	32	64	0.5	48	12	6	0.25	Deletion of nt 432

*CTX* cefotaxime, *CAZ* ceftazidime, *FEP* cefepime, *PIP/TAZ* piperacillin-tazobactam, *ATM* aztreonam, *TOL/TAZ* ceftolozane-tazobactam, *CAZ/AVI* ceftazidime-avibactam, *NA* not applicable, *nt* nucleotide.

**Figure 2:**
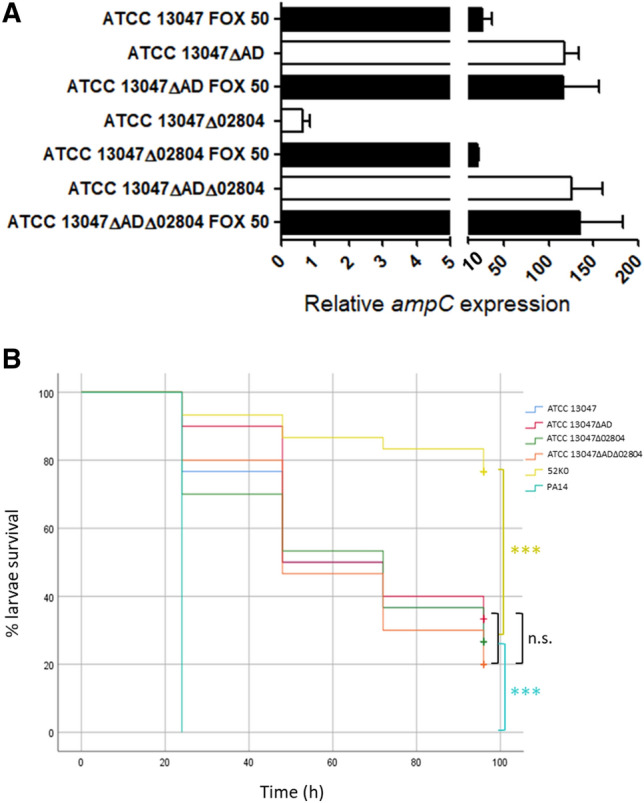
(**A**) Relative quantification of *ampC* (ECL_00553) mRNA in the indicated strains, considering the wildtype ATCC 13047 strain basal level as 1 and using the *rpoB* gene for normalization. Horizontal columns represent the mean values from experimental replicates, whereas the error bars correspond to the SD (linear scale). White columns correspond to mRNA extracted in the basal situation whereas black ones correspond to induction conditions with cefoxitin (FOX) at the indicated concentrations (mg/l). (**B**) Virulence behavior of the indicated strains in the *G. mellonella* infection model. The proportion of surviving larvae after infection with 5E^6^ CFU/worm at the controlled time points (24, 48, 72, 96 h) is shown. Statistical analysis (Kaplan–Meier curves and log-rank test) was performed for pairwise comparisons between all strains. The two controls used in A were *P. aeruginosa* PA14 (high virulence) and *K. pneumoniae* 52K0 (low virulence). ***p value < 0.001; **p value < 0.01; *p value < 0.05; *n.s.* not significant, i.e., *p* value > 0.05.

Since all five *ampD*-defective mutants showed virtually the same resistance phenotype (Table 2), which affected the hydrolizable β-lactams including ceftolozane/tazobactam as previously reported^60^, we selected just one strain for further characterization (denominated ATCC 13047ΔAD for simplification). The phenotype of this mutant was confirmed through real time RT-PCR, showing a value of *ampC* mRNA of ca. 120-fold compared to wildtype strain (Fig. 2A). The ATCC 13047ΔAD hyper-production level, although comparable to that of the cloned β-lactamase in the pUCP24 multicopy vector (ca. 140-fold with regard to wildtype *ampC* expression, Figs. 1A, 2A), provided higher MICs of ceftazidime, cefotaxime, aztreonam, and piperacillin/tazobactam for instance (Tables 1, 2). The explanation could be the reported induction effect exerted by cefotaxime (and presumably other β-lactams) at high concentrations on the expression of the chromosomal *ampC*^31^, already hyperexpressed owing to *ampD* inactivation, a circumstance that would obviously not be affecting the cloned gene. Regardless, the basis for *ampD* inactivation-mediated AmpC hyperproduction has been thoroughly studied in different species, including *E. cloacae*^31^. In regular conditions, AmpD amidase performs a key step for the degradation of cytosolic soluble muropeptides, i.e., cleavage of bonds between the 1,6-anhydro-*N*-acetyl-muramic acid and the l-alanine of the stem peptides, needed for the subsequent synthesis of new peptidoglycan precursors incuding UDP-*N*-acetyl-muramic acid-pentapeptides^11,12,22^. These latter molecules promote the repressor conformation of AmpR keeping *ampC* expression to a minimum. Conversely, when AmpD function is lost (or saturated), the 1,6-anhydro-*N*-acetyl-muramic acid-peptides displace the UDP-*N*-acetyl-muramic acid-pentapeptides from AmpR binding, enabling its activator conformation and AmpC hyper-production^11,12,22^.

Although AmpC hyperproduction-based β-lactam resistance has been deeply studied in *E. cloacae* from almost all perspectives^21,22,25,31,58,59,61–63^, its potentially associated biological cost has never been quantified, which constrasts with awide array of β-lactamases, properly characterized in this sense^14,43,45^. Cloning AmpC in a multicopy plasmid (previous section) enabled us to measure the direct impact of hyperproducing an instrinsic β-lactamase in a similar way as that for acquired enzymes, which usually show constitutive high expression thanks to strong promoters^64,65^. As mentioned above, our results ruled out any virulence attenuation associated with AmpC hyperproduction itself (Fig. 1B). However, this way of obtaining an AmpC hyperproducer strain is obviously artificial, and therefore, we sought to ascertain whether hyperproduction together with its archetypical mutation-driven mechanism (*ampD* disruption)^21,25,27,28,31^ could impact virulence. To answer this question, we performed *G. mellonella* infections with ATCC 13047ΔAD in parallel to the wildtype strain, and as can be seen in Fig. 2B, the dynamics of larvae killing were virtually equal (log rank test *p* value > 0.05 in the pairwise comparison). This result was somehow expectable, since AmpC hyperproduction is probably the most important resistance mechanism by far in clinical strains of *E. cloacae* and other species^25,27,28,45,62^, and thus, it would not be selected that frequently if it caused a high biological cost. Moreover, the AmpD-defective background combines AmpC hyperproduction with a partially impaired peptidoglycan recycling, since this amidase performs a key step to enable an efficient cytosolic anabolism of new peptidoglycan precursors^11,22^. However, not even this combination of features had any impact on *E. cloacae* virulence, as happens for the *P. aeruginosa* AmpD-defective AmpC hyperproducers^13^. This observation supports our abovementioned results in which a virtually complete blockade of peptidoglycan recycling (*ampG* inactivation or even *ampG* inactivation + pUCPAC_EC_) did not affect *G. mellonella* killing either (Fig. 1B). Therefore, *E. cloacae* seems very resistant in terms of its virulence being not affected by cell-wall metabolism disturbance and β-lactamase hyperproduction, at least in comparison with *P. aeruginosa* for instance^16,39^.

### AmpC shows a single step derepression dynamics linked to AmpD inactivation in *E. cloacae*

A very important body of information is available concerning the chromosomal AmpC of *E. cloacae*^4,17–21,63^. However, there are still some knowledge gaps on the topic that we wanted to fill, given the relevance of this ESKAPE pathogen and this resistance mechanism^2,4^. As mentioned above, some classic works reported different types of AmpD alterations causing AmpC hyperproduction in *E. cloacae* and closely related species^21,34–37^. However, because of the use of different species, strains, methodologies/models, and spontaneous mutants, a clear cause-effect relatedness between the type of *ampD* mutation (amino acid changes, frameshifts, stop codons) and the final phenotype of AmpC production (total derepression, partial derepression, hyper-inducibility) cannot be deduced^21,34–37^. Thus, we hypothesized that at least in some of the cases, a double inactivation of *ampD* plus its additional homologue could have gone unnoticed as cause of total AmpC derepression. This idea was deduced from the model described in *P. aeruginosa* and later shown to be conserved in *Yersinia enterocolitica*^9,13,65^, in which the three-step sequential inactivation of *ampD* and its two additional homologues (*ampDh2* and *ampDh3*) causes increasing levels of AmpC production, inducibility and resistance, reaching total derepression with the triple mutant. Moreover, it was demonstrated that this multiplicity of AmpD homologues provides the advantage of enabling greater levels of β-lactam resistance without losing fitness/virulence, attenuation only happening when the three homologues were disrupted^13,14^. Thus, a similar model could exist in *E. cloacae*, in this case with a unique additional *ampD* homologue (ECL_02804 in the strain ATCC 13047). In accordance with previous evidence^13,66^, this amidase was considered the homologue of the periplasmic AmpDh2 of *P. aeruginosa* and AmiD of *Escherichia coli*^13,66^, and potentially key to enable AmpC overproduction in two steps: whereas disruption of AmpD would cause a partial derepression, double *ampD*-ECL_02804 inactivation would entail a maximum production of AmpC (not further inducible). To ascertain whether or not this hypothesis was true, we constructed a simple mutant in the ECL_02804 gene, and the double KO mutant in ECL_02804 and *ampD* using the spontaneous ATCC 13047ΔAD as a scaffold. However, as can be seen in Table 2 and Fig. 2A, neither the inactivation of ECL_02804 in the wildtype strain, nor in the *ampD-*defective background had any significant effect on the expression of *ampC* and resistance profile compared to the respective originative strains. Moreover, ATCC 13047Δ02804 displayed a wildtype inducibility profile, whereas both ATCC 13047ΔAD and ATCC 13047ΔADΔ02804 showed no significant changes in their behavior after cefoxitin challenge either (Fig. 2A). In other words, the level of *ampC* mRNA was similar in basal vs cefoxitin induction conditions in these two AmpD-defective strains, suggesting that inactivation of *ampD *per se is enough to cause a virtually complete AmpC derepression . Therefore, the amidase encoded by ECL_02804 seems to lack any regulator role over *E. cloacae* intrinsic cephalosporinase. This observation would be somehow in accordance with the fact that in *P. aeruginosa* the repressor power of AmpDh2 over AmpC production was shown to be significantly less important than those of AmpDh3 and mostly of AmpD^9^.

Therefore, no stepwise derepression seems to exist for *E. cloacae* AmpC according to our results, although whether or not this could true for other related species harboring intrinsic cephalosporinases (*C. freundii, M. morganii*, and so on) remains to be investigated. Thus, we believe that the intermediate phenotypes of AmpC hyperproduction found in the abovementioned classical works^21,33–37^ could be related to *ampD* mutations leading to an incomplete inactivation of the protein, and/or other neglected mutations/features in the strains/species used. Meanwhile, the inactivation of ECL_02804 alone or in an *ampD*-defective background had no impact on *E. cloacae* virulence either, as can be seen in Fig. 2B. Since ECL_02804 is allegedly performing the same role as *P. aeruginosa* AmpDh2, i.e., cleavage and turnover of stem lateral peptides from the murein sacculus^11,45,66,67^, the double amidase mutant should have a drastically altered peptidoglycan metabolism. Yet not even this situation, added to the derived AmpC derepression, affected the *G. mellonella* killing behavior of *E. cloacae*. These data are in line with the aforementioned idea of the high resistance of this species against peptidoglycan metabolism alterations, which contrasts with other more susceptible microorganisms^16,39,50,56^.

### Analyzing the enigmatic role of the supranumerary putative AmpC homologue in *E. cloacae*

**Figure 3:**
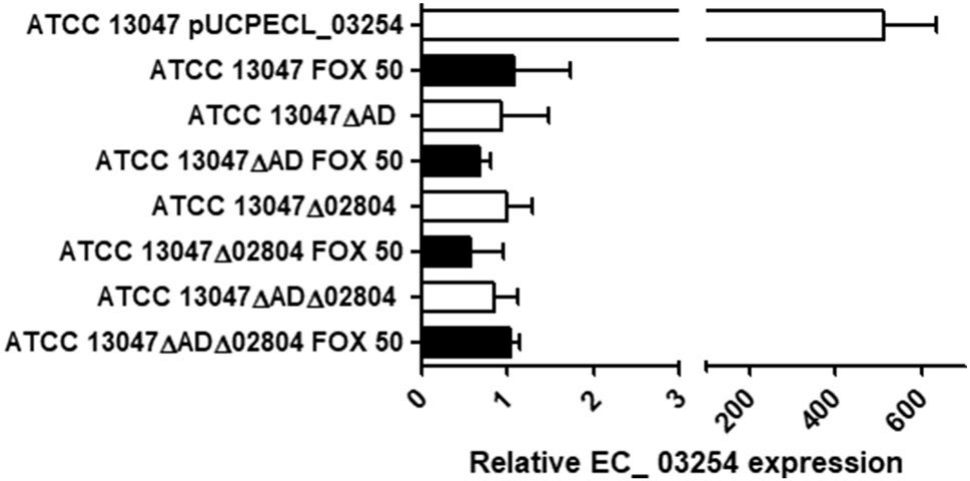
Relative quantification of the ECL_03254 gene mRNA in the indicated strains, considering the wildtype ATCC 13047 strain basal level as 1 and using the *rpoB* gene for normalization. Horizontal columns represent the mean values from experimental replicates, whereas the error bars correspond to the SD (linear scale). White columns correspond to mRNA extracted in basal situation, whereas black ones correspond to induction conditions with cefoxitin (FOX) at the indicated concentrations (mg/l).

The final knowledge gap we wanted to fill dealt with the enigmatic additional class C β-lactamase that is generally encoded together with its own AmpR-type regulator in the genomes of *E. cloacae*^31^. This β-lactamase, encoded by the ECL_03254 gene in the ATCC 13047 strain, has previously been shown to be not inducible through cefoxitin challenge^31^. Moreover, its deletion caused no effects for the wildtype β-lactam susceptibility profile of *E. cloacae* either^31^. Therefore, we hypothesized that this supernumerary AmpC could be contributing to the resistance phenotype not in basal conditions, but in a situation in which the primary AmpC (ECL_00553 gene in the ATCC 13047 strain) is already overproduced through mutational pathways such as AmpD disruption. Thus, the AmpR-activating muropeptides accumulated in this scenario, which could differ from those predominating during cefoxitin challenge as recently proposed^12^, would not only promote the expression of the primary *ampC* but also of the secondary gene through binding to its own AmpR regulator (ECL_03253 in the ATCC 13047 strain). To check this possibility we analyzed the expression of ECL_03254 in the wildtype strain, and in the mutants ATCC 13047ΔAD and ATCC 13047ΔADΔ02804, but its relative mRNA did not significantly change in any case (Fig. 3). These facts indicated that this enigmatic β-lactamase does not contribute to resistance of *E. cloacae* even in a situation of accumulation of AmpR activator muropeptides leading to the regular AmpC (ECL_00553 gene) hyperproduction. Moreover, these data confirmed the lack of any regulatory role of the amidase homologue ECL_02804 over any *E. cloacae* β-lactamase.

On the other hand, we hypothesized that ECL_03254 could perhaps have alternative unknown hyperproduction mechanisms, which would contribute to an increased resistance in *E. cloacae* in specific conditions. In this sense, we cloned the ECL_03254 gene in the pUCP24 multicopy vector obtaining the plasmid pUCPECL_03254 to check whether, once hyper-expressed, this putative cephalosporinase could provide a boosted resistance level. Once transformed in *E. coli* XL1 Blue and in the *E. cloacae* wildtype strain, only very minor increases in cephalosporins MICs were seen (Table 1), although the level of ECL_03254 expression was above 500-fold compared to reference (Fig. 3). Therefore, these results indicate that, although highly conserved in *E. cloacae*, this putative AmpC-type β-lactamase apparently has no relevant effects on phenotype even in a situation of extreme overproduction.

Thus, the enigma as to which role the *E. cloacae* secondary AmpC enzyme and its AmpR-type regulator could play, and why they are so conserved in natural strains remains to be deciphered. This resembles other enzymes widely distributed among natural strains, but apparently not contributing to the resistance of the producing species, such as the carbapenemase PoxB in *P. aeruginosa*^68^. Given the proposed evolutionary origin of β-lactamases from PBPs—enzymes with roles essential for the correct peptidoglycan construction^47,48,53^—it could be argued that this type of enigmatic β-lactamases may have some constitutive activities related to murein sacculus metabolism (e.g., contributing to its remodeling, turnover, fine-tuning of crosslinking, length of sugar chains, etc.), like many other enzymes in the periplasm^69,70^. A potential activity of this type of β-lactamases not over the entire peptidoglycan but in metabolizing soluble fragments to enable cytosolic recycling reactions or even participating in regulatory networks cannot be ruled out either^22,23^. In conclusion, investigating the real activity of enzymes like ECL_03254 even beyond β-lactam resistance is a topic worth delving into so as to understand bacterial biology and find weak points potentially useful to design therapeutic weapons.

### Concluding remarks

Here we provide novel data regarding AmpC-dependent resistance in *E. cloacae,* which matches the knowledge level to that of other pathogens, revealing interesting particularities. Our results point to *E. cloacae* as a pathogen with high resistance to the costs usually associated with β-lactamase hyper-production and/or peptidoglycan alterations in other species. These observations may be carefully considered for the future development of strategies to defeat one of the greatest healthcare challenges of the twenty-first century, as is multi-drug resistant *E. cloacae*.

## Methods

### Bacterial strains, plasmids, and antibiotic susceptibility testing

A general list and description of the laboratory strains and plasmids used in this work are shown in Table S1. In the indicated strains, susceptibility testing to determine the minimum inhibitory concentration (MIC) of representative β-lactams was performed using MIC test strips (Liofilchem) or E-test strips (bioMérieux) following the manufacturer’s instructions. When necessary, Müller–Hinton broth microdilution was performed following standard procedures.

### Cloning of *Enterobacter cloacae* AmpC β-lactamases

To clone the *E. cloacae* ATCC 13047 strain chromosomal AmpC β-lactamase gene (ECL_00553) into the pUCP24 multi-copy vector, the primers shown in Table S2 (AmpC_EC_-F-SacI and AmpC_EC_-R-BamHI) were used with the ATCC 13047 strain’s DNA as a template. The PCR products obtained were purified, digested, and ligated into the linearized vector. The resulting plasmid (pUCPAC_EC_) was transformed into *E. coli* XL1 Blue through the CaCl_2_ heat-shock method. After extraction of plasmids through commercial kits, they were electroporated into the *E. cloacae* strains indicated following standard protocols, and constructs were checked for the absence of mutations through Sanger sequencing (Macrogen) with the abovementioned primers. To clone the putative supranumerary AmpC-type β-lactamase of *E. cloacae* (gene ECL_03254 of ATCC 13047 strain), the same procedures were followed. The primers shown in Table S2 were used, finally obtaining the plasmid pUCPECL_03254 that was transformed into *E. coli* XL1 Blue and *E. cloacae* ATCC 13047 to analyze the phenotypes obtained (MIC determination and RT-PCR of ECL_03254 gene). Absence of mutations in pUCPECL_03254 was also checked as mentioned above.

### Analysis of gene expression

The mRNA of the indicated genes in the corresponding strains was quantified through real-time reverse transcription PCR (RT-PCR) and specific primers (Table S3), according to previously described protocols^14^. Briefly, total RNA proceeding from exponential phase cultures was extracted with the RNeasy minikit (Qiagen) and treated with 2 U of Turbo DNase (Ambion) for 60 min at 37 °C to remove contaminating DNA. Then 50 ng of purified RNA were used for real-time RT-PCR using the QuantiTect SYBR Green RT-PCR Kit (Qiagen) in a CFX Connect device (Bio-Rad). Previously described primers for the *rpoB* housekeeping gene were used to normalize mRNA levels^71^ (Table S3), and the results for the genes of interest were referred to the indicated control strain in each case, being expressed as relative values. All real time RT-PCRs were performed in duplicate, and mean values of expression from three independent RNA extractions were considered. For induction experiments, the corresponding cultures were exposed to cefoxitin for 2.5 h prior to RNA extraction. Concentration of cefoxitin depended on the strain (see “Results and discussion” section), but a general rule of using concentrations between 1/4 and 1/8 of the MIC of the corresponding strain was applied, following previous works in which these and even lower concentrations were shown to significantly induce the different β-lactamases^40,41^.

### Construction/obtaining of mutants

To inactivate the different genes indicated, the protocol of Huang and coworkers was followed^72^. Briefly, the FRT (flippase recognition targets)-flanked apramycin resistance cassette (*aac(*3*)IV*) was amplified using the plasmid pMDIAI (Table S1) as a template and the corresponding specifically-designed primers, i.e., containing fragments of ca. 60–80 nucleotides homologous to the target gene as tails preceding the nucleotides complementary to the FRT sites (Table S2). The obtained amplicons were electroporated into the *E. cloacae* ATCC 13047 strain that had been previously transformed with the pACBSR-Hyg plasmid. This vector contains an arabinose-inducible recombinase enabling homologous recombination between the chromosomal gene and the electroporated amplicon. After curation of the latter plasmid, the obtained colonies were checked by PCR and sequencing to confirm the substitution of the wildtype gene by the apramycin resistance gene flanked by FRT sites and the abovementioned 60–80 nucleotide tails. Finally, when needed to eliminate the apramycin resistance cassette, the mutants were transformed with the plasmid pFLP-hyg that contains the flipase mediating the excision of the *aac(*3*)IV* gene after overnight incubation at 43 °C. After curation of the latter plasmid, the candidate colonies were checked by PCR and Sanger sequencing (Macrogen). All the plasmids and primers used to carry out this protocol are displayed in Tables S1 and S2.

Spontaneous chromosomal AmpC β-lactamase hyperproducer mutants were obtained by plating different dilutions of ATCC 13047 strain overnight cultures in LB agar plates supplemented with cefotaxime 8 mg/l. Selected cefotaxime-resistant colonies were characterized through β-lactam MIC determination and real time RT-PCR of *ampC*. The mutational inactivation of *ampD* gene has been described as the archetypical mechanism enabling hyperproduction of *E. cloacae* AmpC^25,27,28,31,35^, and therefore, selected candidate colonies were checked through PCR/Sanger sequencing (Macrogen) with specific primers for this gene (Table S2).

### Invertebrate infection model

The wax moth *G. mellonella* was used as the infection model following previously described protocols with minor modifications^14,73^. Exponentially growing cultures of the corresponding strains were pelleted, washed, and resuspended in Dulbecco’s phosphate buffered saline without calcium/magnesium (PBS, Biowest). Different serial dilutions (1E^9^ colony forming units, CFU/ml to 1E^6^ CFU/ml) were made in PBS and injected using Hamilton syringes (10-µl aliquots) into individual *G. mellonella* larvae (approx. 2–2.5 cm long caterpillars weighing 200–300 mg) via the hindmost left proleg. Ten larvae were injected for each dilution and strain and scored as live or dead after 24, 48, 72, and 96 h at 37 °C. These preliminary assays were carried out to choose the appropriate dose of 5E^6^ CFU/larva that was used to analyze survival through Kaplan–Meier curves and log-rank tests (considering a p value < 0.05 as significant in the pairwise comparisons), compiling the data obtained from at least three independent replicates. The 5E^6^ CFU/larva inoculum was chosen because it was the one showing the greatest differences in larvae killing capacity between strains, with step-wise dynamics at the different time points in the assay.

### Statistical analysis

With the exception of Kaplan Meier curves/log rank test (SPSS software, version 25.0) and Probit model (R software, version 3.2.2), GraphPad Prism 7 was used for statistical analysis and graphical representation. Quantitative variables were analyzed through one-way ANOVA (with Tukey’s post-hoc multiple comparison test) by pairing data obtained from the experimental replicates (i.e. matched observations), and/or Student’s t test (two-tailed, paired), as appropriate. A p value < 0.05 was considered statistically significant.

### Supplementary Information

Supplementary Tables.

## Data Availability

The data generated for this study are available upon request to the corresponding authors.
