# Supplementary material for: Filling knowledge gaps related to AmpC-dependent β-lactam resistance in Enterobacter cloacae
Source: Sci Rep. 2024 Jan 2;14:189. doi: 10.1038/s41598-023-50685-1 (PMC10762043; doi:10.1038/s41598-023-50685-1)
Supplement: Supplementary file 1 — Supplementary Tables. [file 41598_2023_50685_MOESM1_ESM.docx]

**SUPPLEMENTARY INFORMATION**

**Table S1.** Strains and plasmids used in this work and their relevant features

| **Strain or plasmid** | **Genotype/characteristics** | **Reference or source** |
| --- | --- | --- |
| *Enterobacter cloacae* | | |
| ATCC 13047 | *E. cloacae* subsp. *cloacae* ATCC 13047 type strain | American Type Culture Collection (ATCC) |
| ATCC 13047ΔAG | *ampG* (ECL_01191) knockout mutant derived from ATCC 13047. *ampG* encodes the permease enabling the entry of most soluble peptidoglycan fragments into cytosol for recycling and AmpC β-lactamase regulation. | This work |
| ATCC 13047ΔAD | *ampD* (ECL_00906) anhydro-N-acetylmuramyl-peptide amidase-disrupted spontaneous mutant obtained from ATCC 13047 (frameshift mutation: deletion of nucleotide 432). Since AmpD works as a cytosolic indirect *ampC* repressor, this strain shows β-lactamase hyperproduction | This work |
| ATCC 13047Δ02804 | ECL_02804 knockout mutant derived from ATCC 13047. ECL_02804 codifies for a periplasmic N-acetylmuramyl-peptide amidase homologous to *Pseudomonas aeruginosa* AmpDh2 | This work |
| ATCC 13047 ΔADΔ02804 | ECL_02804 knockout mutant derived from ATCC 13047ΔAD; therefore it is a double knockout mutant for the indicated genes | This work |
| *Pseudomonas aeruginosa* | | |
| PA14 | Highly virulent and cytotoxic reference strain, used as a control for high virulence in the *G. mellonella* infection model | [1] |
| *Klebsiella pneumoniae* | | |
| 52K0 | CPS expression-defective mutant derived from Kp52.145 strain, used as a control for attenuated virulence in the *G. mellonella* infection model | [2] |
| Strains and plasmids used for cloning, construction and complementation of knockout mutants | | |
| *Escherichia coli* XL1 Blue | Strain used as a host for replication and storage of different plasmids | [3] |
| pUCP24 | Gm^r^; pUC18-based multicopy shuttle vector | [4] |
| pUCPAC_EC_ | Gm^r^; pUCP24 containing the *E. cloacae* ATCC 13047 strain *ampC* (ECL_00553) wild-type gene | This work |
| pUCPECL_03254 | Gm^r^; pUCP24 containing the *E. cloacae* ATCC 13047 strain putative additional AmpC-type enzyme (ECL_03254) wild-type gene | This work |
| pMDIAI (Addgene #51655) | pMD18-T simple backbone containing an apramycin resistance gene (*aac(3)IV*) flanked by FRT sites; template for amplification of this resistance cassette | Addgene; [5] |
| pACBSR-Hyg (Addgene #87830) | A p15A replicon plasmid containing an arabinose-inducible λ-Red recombinase and a hygromycin resistance marker | Addgene; [6] |
| pFLP-Hyg (Addgene #87831) | Plasmid bearing a heat-shock inducible FLP recombinase and a hygromycin resistance marker | Addgene; [6] |

Abbreviations: Apra^R^: apramycin resistance cassette; CPS: capsule polysaccharide; FRT: flippase recognition target; FLP: flippase recombinase; Gm^r^: gentamicin resistant; Kan^r^: kanamycin resistant; Rif^r^: rifampin resistant; ST: sequence type; Str^r^: streptomycin resistant; kb: kilobase.

**Table S2.** Primers designed in this work for cloning, sequencing, and construction of knockout mutants

| **Primer** | **Sequence (5′-3*′*)^a,b^** | **PCR size (bp)** | **Application** |
| --- | --- | --- | --- |
| AmpC_EC_-F-SacI | CGC**GAGCTC**GCGGACTCGCTATTACGGAAGATAAC | 1195 | Cloning and sequencing of *ampC* (ECL_00553) from *E. cloacae* ATCC 13047 |
| AmpC_EC_-R-BamHI | CGC**GGATCC**GCGTTACTGTAGCGCGTCGAGG |  |  |
| ECL_03254-F-SacI | ATA**GAGCTC**ATACCTTCCTGAACCAAACGAGATAA | 1324 | Cloning and sequencing of putative additional AmpC–type enzyme (ECL_03254) from *E. cloacae* ATCC 13047 |
| ECL_03254-R-BamHI | CGC**GGATCC**GCGTCAGCGCAGCGCCTGTGCAA |  |  |
| EC_AG_KO_F | ATGTCCAGTCATTACTTACGCATTTTCCAGCAACCCAAATCAGCCATTCTGCTGATCCTTATTCCGGGGATCCGTCGACC | 1491 | Amplification of apramycin resistance gene with added 60-nt tails corresponding to upstream and downstream sequences of *E. cloacae* ATCC 13047 *ampG,* to enable homologous recombination |
| EC_AG_KO_R | TCAGGTTGTCTGCGTTTTACGCAGAGCCAGAAAATCGAGCAGCCCACCGAGCAGGATCCCTGTAGGCTGGAGCTGCTTC |  |  |
| EC_02804_KO_F | TAAAGATGCCAGCATGCTGATGGTGAGCGTAAGCGGTACGGCGGTGAAAACCCGTCGATGATTCCGGGGATCCGTCGACC | 1491 | Amplification of apramycin resistance gene with added 60-nt tails corresponding to upstream and downstream sequences of *E. cloacae* ATCC 13047 ECL_02804, to enable homologous recombination |
| EC_02804_KO_R | AGATTAATCTTATGATCATGCTCACAAAAACGAGGCATTCGGGTTAATCCCGAAGGATTATGTAGGCTGGAGCTGCTTC |  |  |
| EC AD-F | GCTACTCTGAACCGAGTAAC | 650 | PCR and sequencing of *E. cloacae* ATCC 13047 *ampD* gene (ECL_00906) |
| EC AD-R | AAGCGTTCTGCGATGATGAC |  |  |

^a^Restriction sites in primers used for cloning are shown in bold. ^b^ In the primers used for gene inactivation, the nucleotides corresponding to the FRT sites flanking the apramycin resistance cassette are underlined. Meanwhile, the rest of the sequences of this type of primers correspond to ca. 60-80 nucleotide fragments upstream/downstream (F/R primers respectively) of each gene to be inactivated.

**Table S3.** Primers used for the analysis of gene expression by real time RT-PCR

| **Primer** | **Sequence (5′-3*′*)** | **Target and amplicon size (bp)** | **Reference or source** |
| --- | --- | --- | --- |
| EC_rpoB_F | CAGCCGCGAACAGGTTGACTACA | *E. cloacae* ATCC 13047 strain *rpoB* housekeeping gene (63 bp) | [7] |
| EC_rpoB_R | GACGCACCGACGGATACCACCTG |  |  |
| EC_ampC_F | ACGCGTCTGTACGCCAACACCAG | *E. cloacae* ATCC 13047 strain *ampC* (ECL_00553) gene (64 bp) | This work |
| EC_ampC_R | TGCCGGACGGTTTAACGGCCAGT |  |  |
| EC_ 03254_F | TGGCGGCGTACCTGGAGAAAAGCA | *E. cloacae* ATCC 13047 strain putative *ampC* family gene (ECL_03254) (62 bp) | This work |
| EC_ 03254_R | CCATACGCCGTCGTTCGCCATCC |  |  |

**SUPPLEMENTARY INFORMATION REFERENCES**

1. Lee, D.G. *et al*. Genomic analysis reveals that Pseudomonas aeruginosa virulence is combinatorial. *Genome Biol*. **7,** R90. https://doi.org/10.1186/gb-2006-7-10-r90 (2006).
2. Cortés, G., Alvarez, D., Saus, C. & Albertí, S. Role of lung epithelial cells in defense against *Klebsiella pneumoniae* pneumonia. *Infect. Immun*. **70**, 1075-1080. <https://doi.org/10.1128/IAI.70.3.1075-1080.2002> (2002).
3. Bullock, W.O., Fernandez, J.M. & Short J.M. XL1 Blue: a high efficiency plasmid transforming *recA Escherichia coli* strain with beta-galactosidase selection. *BioTechniques* **5**, 376-379 (1987).
4. West, S.E., Schweizer, H.P., Dall, C., Sample, A.K. & Runyen-Janecky, L.J. Construction of improved *Escherichia-Pseudomonas* shuttle vectors derived from pUC18/19 and sequence of the region required for their replication in *Pseudomonas aeruginosa*. *Gene* **148**, 81-86. <https://doi.org/10.1016/0378-1119(94)90237-2> (1994).
5. Yang, J. *et al*. High-efficiency scarless genetic modification in *Escherichia coli* by using lambda red recombination and I-SceI cleavage. *Appl. Environ. Microbiol*. **80**, 3826-3834. https://doi.org/10.1128/AEM.00313-14 (2014).
6. Huang, T.W. *et al*. Capsule deletion via a λ-Red knockout system perturbs biofilm formation and fimbriae expression in *Klebsiella pneumoniae* MGH 78578. *BMC Res Notes* **7**,13. https://doi.org/10.1186/1756-0500-7-13 (2014).
7. He, G.X. *et al*. SugE, a new member of the SMR family of transporters, contributes to antimicrobial resistance in *Enterobacter cloacae*. *Antimicrob. Agents Chemother*. **55**, 3954-3957. https://doi.org/10.1128/AAC.00094-11 (2011).
